# Burden of enterotoxigenic *Escherichia coli* and shigella non-fatal diarrhoeal infections in 79 low-income and lower middle-income countries: a modelling analysis

**DOI:** 10.1016/S2214-109X(18)30483-2

**Published:** 2019-02-14

**Authors:** John D Anderson, Karoun H Bagamian, Farzana Muhib, Mirna P Amaya, Lindsey A Laytner, Thomas Wierzba, Richard Rheingans

**Affiliations:** a Goodnight Family Department of Sustainable Development, Appalachian State University, Boone, NC, USA; b Emerging Pathogens Institute, Gainesville, FL, USA; c Department of Environmental and Global Health, University of Florida, Gainesville, FL, USA; d Bagamian Scientific Consulting, Gainesville, FL, USA; e PATH, Washington, DC, USA

## Abstract

**Background:**

Enterotoxigenic *Escherichia coli* (ETEC) and shigella are two major pathogens that cause moderate-to-severe diarrhoea in children younger than 5 years. Diarrhoea is associated with an increased risk of stunting, which puts children at risk of death due to other infectious diseases.

**Methods:**

We modelled ETEC-related and shigella-related mortality and the effect of moderate-to-severe diarrhoea episodes to determine the number of children with stunting due to these infections in 79 low-income and lower middle-income countries. We applied population attributable risk for increased number of deaths due to other infectious diseases in children who are stunted. We calculated 95% uncertainty intervals (UIs) for the point estimates.

**Findings:**

In children younger than 5 years, we estimate 196 million (95% UI 135–269) episodes of ETEC and shigella diarrhoea occur annually, resulting in 3·5 million (0·8–5·4) cases of moderate-to-severe stunting and 44 400 (29 400–59 800) total ETEC deaths and 63 100 (44 000–81 900) total shigella deaths in 2015. Additional infectious disease mortality due to stunting resulted in increases of 24% (8–34; for ETEC) and 28% (10–39; for shigella) over direct deaths due to diarrhoeal episodes. The distribution of mortality and morbidity varied geographically, with African Region and Eastern Mediterranean Region countries bearing the greatest burden.

**Interpretation:**

The expanded effects of non-fatal ETEC and shigella-related diarrhoeal episodes can have lasting consequences. Prevention of these infections could reduce the risk of direct death and stunting and deaths due to other infectious diseases. Understanding the countries and populations with the highest disease risk helps to target interventions for the most vulnerable populations.

**Funding:**

The Bill & Melinda Gates Foundation.

## Introduction

Diarrhoea mortality has declined significantly globally and in the poorest countries, whereas diarrhoea morbidity has declined more slowly.^1^, ^2^ These observed patterns have refocused attention on the positive relationship between recurring and frequent non-fatal diarrhoea episodes and childhood stunting.^3^, ^4^ Childhood stunting increases the likelihood of death from diarrhoea, pneumonia, and measles, among other infections.^5^ Stunting also affects cognitive development^6^ which might determine a child's ability to learn, their educational attainment, and future earnings.^5^, ^7^, ^8^

Although rotavirus accounts for most diarrhoea-related childhood deaths, other enteric pathogens play an important role in childhood mortality and morbidity.^9^ The Global Enteric Multicenter Study (GEMS) conducted in Africa and Asia found that four diarrhoeal pathogens—rotavirus, shigella, enterotoxigenic *Escherichia coli* (ETEC), and *Cryptosporidium* spp—were significantly associated with moderate-to-severe diarrhoea, accounting for 70% of diarrhoeal disease in children younger than 5 years.^10^

Antibiotic resistance is a growing problem for the treatment of these pathogens.^11^ Shigella is resistant to several antibiotic classes, and several nations report greater than 50% resistance to certain antibiotic classes by pathogenic *E coli*.^11^ This situation makes new prevention strategies critical. Promising interventions under development are vaccines against ETEC and shigella, which can potentially prevent infection, morbidity, and mortality from these pathogens and combat antibiotic resistance by reducing antibiotic use.

Quantification of disease burden, which is a WHO priority research area, is necessary to fully understand and describe the effect of ETEC and shigella vaccines.^12^ In this modelling analysis, we aimed to quantify the disease burden of ETEC and shigella by estimating the number of deaths and disability-adjusted life-years (DALYs) lost over 1 year in children younger than 5 years. We also estimated the burden associated with stunting caused by these infections in children, focusing on the effect of stunting on increased mortality from other childhood infectious diseases (appendix). We explored disease burden across the six WHO global regions by aggregating estimates from 79 low-income and lower middle-income countries (LMICs). Quantifying burden distributions across and within countries provides critical information to policy makers as vaccines are developed.

**Research in context**

**Evidence before this study**

We used two search strings for the literature review; one for the aetiology in children younger than 5 years and another for diarrhoea and stunting. First, we searched PubMed for articles published between Oct 29, 2010, and Oct 29, 2015, without language or publication restrictions by location. We found 59 results for “aetiology under five” with the search string (“ETEC” OR “enterotoxigenic *E coli”* OR “enterotoxigenic” OR “shigella”) AND (“children under five” OR “children under 5” OR “children <5”) AND (“morbidity” OR “illness” OR “stunting” OR “episodes” OR “events” OR “cases”) AND (“cause” OR “etiology” OR “aetiology”). Second, we searched PubMed for articles published between inception and Oct 29, 2015, without language or publication restrictions by location. We found 129 results for “diarrhoea and stunting” with the search string (“diarrhea” OR “diarrea” OR “diarrhoea”) AND (“stunting” OR “height for age” OR “height” OR “Z score”) AND (“child*” OR “children under five” OR “children under 5” OR “children <5”) AND (“outcomes” OR “changes”). From the articles identified, we selected the most relevant peer-reviewed journal articles on the subject of ETEC and shigella deaths in children under 5 years of age, incidence, and the association of stunting and diarrhoea. No publications systematically attempted to quantify the burden of ETEC and shigella diarrhoea together with associated ETEC- and shigella-induced stunting and resulting deaths from other infectious disease. The Global Burden of Disease study has produced estimates of ETEC and shigella diarrhoeal burden and has published estimates of disability-adjusted life-years (DALYs) associated with acute diarrhoea and additional DALYs associated with long-term consequences of undernutrition associated with diarrhoea.

**Added value of this study**

Our study provides a systematic set of estimates of the effect of ETEC and shigella-related diarrhoea on stunting, the associated mortality due to other infectious disease in children who are stunted, and the effect in DALYS in low-income and lower-middle income countries. Our study includes a more nuanced evaluation of disease by quantifying the expanded effects that diarrhoea has on other aspects of a child's development, which might increase a child's risk for death.

**Implications of all the available evidence**

Our estimates suggest that the burden of ETEC and shigella diarrhoea is far greater than previously assumed. Prevention of ETEC and shigella diarrhoea could prevent stunting in vulnerable children and reduce the number of deaths due to other infectious diseases. When evaluating interventions that can prevent these infections, it will be important to consider the full burden of disease as the expanded effect of vaccination could result in many more lives saved.

## Methods

### Study design and population

We selected 84 low-income and lower middle-income countries on the basis of World Bank income categories.^13^ We excluded countries with fewer than ten childhood diarrhoeal deaths per year in children younger than 5 years or countries without data on stunting within past 20 years. We calculated estimates nationally and aggregated estimates into WHO regions to present trends at a regional scale. National population estimates were from 2015 estimated by the UN Population Division.^14^ Table 1 shows all key inputs and assumptions. Hereafter, children are individuals younger than 5 years.

**Table 1:** Key assumptions and inputs in calculating ETEC and shigella burden

	**Values**	**Range**	**Source**	**Reference**
Baseline population estimates for children younger than 5 years	Varies by country	Not included in uncertainty analysis	UN Population Division estimates for 2015	14
Diarrhoeal mortality estimates	Differs in overall deaths and per country	90–110%; triangular*	Midpoint estimate between 2015 MCEE and GBD	15, 16
Aetiological fraction attributed to ETEC by culture for children younger than 5 years, by WHO region	0·043–0·074	75–125%; triangular*	Systematic review; updated with GEMS molecular re-analysis estimates	17
Aetiological fraction attributed to shigella by culture for children younger than 5 years, by WHO region	0·001–0·119	75–125%; triangular*	Systematic review; updated with GEMS molecular re-analysis estimates	17
ETEC molecular adjustment	1·5	±0·5; triangular*	GEMS re-analysis study	18
Shigella molecular adjustment	2·0	±0·5; triangular*	GEMS re-analysis study	18
All-cause diarrhoeal episodes	2·2–3·3 episodes per child-year	80–120%; triangular*	Fischer-Walker and colleagues	19
Caretakers sought care at a health facility after the child experienced an episode of diarrhoea	47%	44–58%; not varied in uncertainty analysis	Demographic and Health Surveys	20
Fraction of diarrhoeal episodes that are moderate to severe	10%	2–15%; triangular*	GEMS Study and Demographic Health Survey	10, 20
HAZ shift with ETEC episodes	0·068	80–120%; triangular*	GEMS estimate table 6	10
HAZ shift for shigella episodes	0·082	80–120%; triangular*	GEMS estimate table 6	10
Rotavirus vaccination adjustment	Varies by country that has introduced rotavirus vaccination	With or without adjustment in sensitivity analysis	Tate and colleagues	21

Ranges for estimates reflect ranges of values varied for uncertainty analysis, unless otherwise noted in the table. The rotavirus vaccination adjustment refers to accounting for the effect of the introduction of rotavirus vaccination in countries before 2014. MCEE=Maternal and Child Epidemiology Estimation Group. GBD=Global Burden of Disease Study. ETEC=enterotoxigenic *Escherichia coli*. HAZ=height-for-age Z score.

* Triangular distributions are continuous with a probability function defined by minimum, maximum, and peak values.

### Diarrhoeal disease burden

#### Aetiological fraction

The disease burden of ETEC and shigella depends upon the fraction of episodes and deaths attributable to each.^10^, ^22^ The application of contemporary molecular approaches has resulted in increased detection of diarrhoeal pathogens^23^, ^24^, ^25^ and development of adjustment factors to refine aetiological disease fractions.^10^, ^18^ However, most existing diagnostic results are from less sensitive culture-based methods. Here, we calculated our aetiological estimates on the basis of culture-based detection methods, but we applied an adjustment factor that takes the under-detection of culture versus molecular diagnostics into account. We applied adjustment factors of 1·5 (50% increase) for ETEC and 2·0 (100% increase) for shigella from the reanalysis of GEMS data^18^ to regional culture-based estimates of both pathogens, as outlined by Lanata and colleagues.^17^ Our approach is conservative with regards to the overall burden of heat-stable ETEC (ST-ETEC) burden. Inclusion of the STp enterotoxin genotype in the GEMS reanalysis resulted in an increased (by 15%) attributable ST-ETEC incidence,^18^ which was not included in the adjustment factor.

We assumed that aetiology does not differ between the causes of moderate-to-severe episodes and mortality. Although data on aetiology of childhood diarrhoeal deaths is scarce, GEMS provided estimates associating a pathogen with moderate-to-severe diarrhoea and the hazard ratio for mortality by diarrhoeal aetiology.^10^ In GEMS, ETEC expressing only heat-labile ETEC (LT-ETEC) was not associated with moderate-to-severe diarrhoea in any age stratum. However, the presence of ST-ETEC (ETEC expressing ST alone or with LT toxin) was associated with elevated mortality risk in the first year of life, but mortality risk for ST-ETEC and shigella were statistically similar to other causes of moderate-to-severe diarrhoea during the second year of life.

#### Mortality and morbidity

There are two sources of diarrhoea mortality estimates: Institute for Health Measurement and Evaluation^15^ and Maternal and Child Epidemiology Estimation Group led by WHO.^16^ We used a mid-point estimate for each country using the 2015 estimates from both organisations to obtain the most accurate data on diarrhoeal deaths per 100 000 children. These results were then apportioned into ETEC and shigella deaths using adjusted aetiological fractions. Diarrhoeal episodes were attributed to ETEC and shigella by multiplying the regional aetiological fractions by regional diarrhoeal disease incidence, as described by Fischer Walker and colleagues (defined as clinically relevant diarrhoea with three or more loose or liquid stools in a 24-h period).^19^

The aetiological fractions of ETEC and shigella were calculated from countries before rotavirus vaccine introduction. However, our diarrhoea mortality estimates included countries that introduced rotavirus vaccines starting in mid-2000s. If we applied pre-introduction ETEC and shigella aetiological fractions to estimates from these countries, we would underestimate ETEC and shigella mortality because vaccination-associated reductions in rotavirus mortality would increase aetiological fractions. Therefore, we adjusted diarrhoeal mortality estimates with rotavirus mortality fractions before and after rotavirus vaccination, as per data from Tate and colleagues.^21^ The adjustment is applied to countries that introduced rotavirus vaccine before 2014, and the effect is explored in sensitivity analysis by excluding adjustments. Adjusted mortality estimates (*m*ʹ) were calculated for each applicable country using (1), where *r* equalled the fraction of rotavirus mortality before vaccination, *r*ʹ was the post-vaccination fraction, and *m* was the current mortality estimate.

(1) $$m'=\frac{m}{(1-r)+r'}$$

### Mortality associated with stunting induced by childhood diarrhoea

Childhood diarrhoeal events can reduce linear growth and increase stunting.^3^, ^26^ We modelled the potential increase in number of children with stunting due to ETEC and shigella by country. We used this output to calculate deaths due to other infectious diseases for which stunting is a risk factor.

In a pooled analysis from all GEMS sites, Kotloff and colleagues^10^ reported differential height-for-age Z score (HAZ) shifts caused by moderate-to-severe diarrhoea cases for children in different age categories. We integrated these data into our calculations of the differential effect of ETEC and shigella on stunting. We calculated the mean fraction of moderate-to-severe diarrhoea cases occurring in three age groups (<12, 12–23, and 24–59 months) and applied the mean HAZ differences (between cases and controls) to the corresponding age group. The average shift of a moderate-to-severe diarrhoea case was 0·068 HAZ for ETEC and 0·082 HAZ for shigella. We assumed that this shift affects the entire population, because a recent multi-level analysis reported moderate-to-severe diarrhoea-related declines in average HAZ scores across the entire child population, not just the most at-risk children.^27^

To estimate the proportion of diarrhoeal episodes considered moderate-to-severe in the population and sufficient to cause a shift in HAZ, we assumed 22% of all diarrhoeal episodes where care was sought at a health facility were moderate-to-severe, on the basis of GEMS findings.^10^ We adjusted this value by calculating the proportion of children who experienced diarrhoea 2 weeks before the survey^20^ and had caretakers that sought care for diarrhoea at a health facility. For countries without Demographic and Health Survey estimates of health-care seeking behaviour, we substituted the mean percentage of health-care seeking of the other countries in the same WHO region. We estimated 47% to be the average fraction of children whose caretakers sought care at a health facility after the child experienced an episode of diarrhoea (table 1). We then assumed that 22% of those children presented with moderate-to-severe diarrhoea, on the basis of GEMS estimates.^10^ Thus, we deduced that 10% (22% × 47%) of child diarrhoeal episodes were moderate-to-severe episodes in each region.

We estimated the effect of this marginal shift in HAZ on the fraction (and number) of children who are categorised as moderately and severely stunted. We used recent Demographic and Health Survey^20^ and WHO^28^ national estimates of the proportion of children classified as moderately stunted (HAZ between −2 and −3) and severely stunted (HAZ less than −3). We fit the full normal distribution of HAZ (*P*) using national estimates of moderate and severe stunting in children as values at −2 and −3 standard deviations below the mean (*μ* [2]). We then calculated a second, hypothetical distribution (*P*') where means are shifted (∆) by 0·068 for ETEC or 0·082 for shigella to the right (*μ*' [3]), simulating a distribution without the effects of moderate-to-severe diarrhoea episodes on stunting for each pathogen. Although ETEC and shigella episodes occur among specific children, this approach simulates an average expected shift among all children. We calculated the differences in probabilities of moderate stunting (*Y*_m_) and severe stunting (*Y*_s_) between the two distributions in (4) and (5), using the cumulative distribution function.

(2) $$P∼N(\mu ,\sigma^{2})\text{where}\sigma =1$$

(3) $$P'∼N(\mu ',\sigma^{'2})\text{where}\mu '=\mu +\Delta \text{and}\sigma '=1$$

(4) $$Y_{m}=[P(X<-2\sigma ')-P'(X'<-2\sigma ')]$$

(5) $$Y_{s}=[P(X<-3\sigma )-P'(X'<-3\sigma ')]$$

These values are used to estimate the increased percentage and number of children classified as moderately and severely stunted by HAZ.

Moderate and severe stunting are risk factors for mortality owing to pneumonia, malaria, measles, and diarrhoeal disease.^29^ The population attributable risk (PAR) approach is used to estimate the fraction of deaths from infectious diseases attributable to excess stunting induced by ETEC and shigella episodes. The risk factor proportion (*P*_e_) was the fraction of children with ETEC-induced or shigella-induced moderate and severe stunting. We used the relative mortality risk associated with different levels of stunting, as outlined by Black and colleagues (*RR*_e_ [6]).^29^ For each of these IDs, we used the PAR equation to estimate the fraction of deaths due to ETEC-induced or shigella-induced stunting in each country.

(6) $$PAR=P_{e}(RR_{e}-1)/[1+P_{e}(RR_{e}-1)]$$

We combined the PARs with national under-five mortality estimates for each of these causes to estimate deaths due to measles, pneumonia, and malaria.^29^ For diarrhoeal disease, we used Maternal and Child Epidemiology Estimation Group and Global Burden of Disease mortality estimates. To avoid double counting, we only used this approach for diarrhoea-associated mortality caused by pathogens other than ETEC and shigella. We assumed that the increased risk of ETEC-induced and shigella-induced stunting only occurs among children younger than 5 years.

### DALY calculations

We converted ETEC and shigella mortality data into DALYs using age weighting and discounting.^30^, ^31^ Years lived with disability was calculated using an episode duration of 0·2 and disability weight of 0·021,^31^ and years of life lost was calculated using standard life expectancy.^32^ For stunting-induced deaths from infectious diseases, only years of life lost are included in DALY calculations because we were unable to estimate infectious disease-related morbidity.

### Sensitivity and uncertainty analysis

We did a series of analyses assessing the effect of uncertainty on the predicted outcomes in each WHO region. We characterised key input variables as distributions (table 1). We used one-way sensitivity analyses in Sensit (version 1.53; TreePlan Software, San Francisco, CA, USA) to estimate the effect of changes in individual input values on the distribution of ETEC and shigella deaths (direct and because of stunting). We assessed uncertainty in our model using Monte Carlo simulations in SimVoi (version 3.07; TreePlan Software). We did 10 000 iterations of our model and generated 95% uncertainty intervals (95% UIs) for key outputs.

### Role of the funding source

The funder of the study had no role in study design, data collection, data analysis, data interpretation, or writing of the report. The corresponding author had full access to all the data in the study and had final responsibility for the decision to submit for publication.

## Results

From 84 LMICs screened on the basis of World Bank income categories, we excluded five countries; 79 countries were included (appendix).

**Figure 1:**
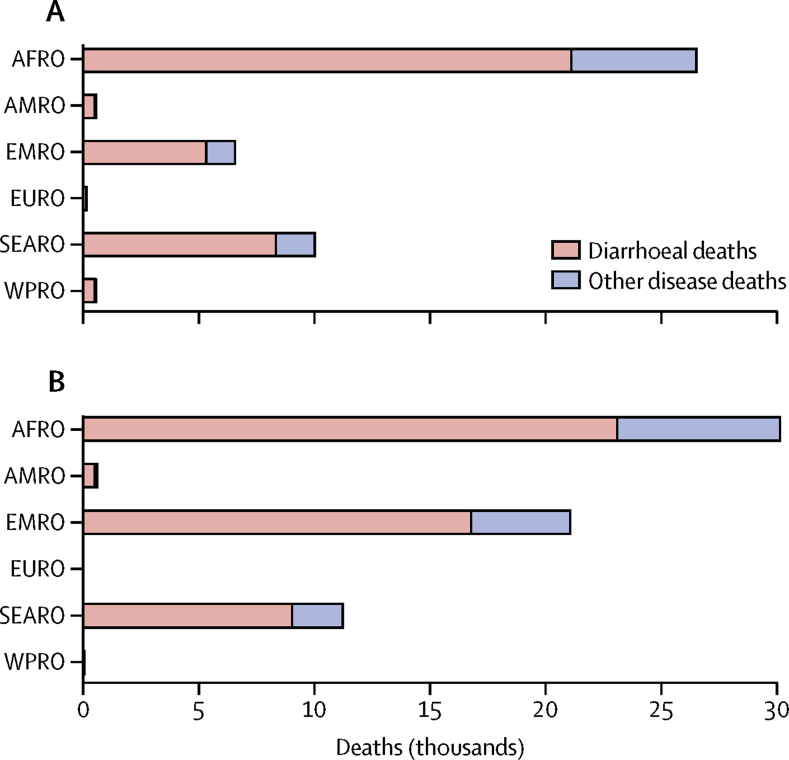
Mortality due to enterotoxigenic *Escherichia coli* (A) and shigella (B) diarrhoea and other infectious diseases in children younger than 5 years, by WHO region Other infectious diseases are all other deaths from infections caused by enterotoxigenic *Escherichia coli*-induced and shigella-induced stunting. AFRO=African Region. AMRO=Region of the Americas. EMRO=Eastern Mediterranean Region. EURO=European Region. SEARO=Southeast Asian Region. WPRO=Western Pacific Region.

ETEC diarrhoea and deaths from ETEC-induced stunting caused an estimated 44 400 (95% UI 29 400–59 800) deaths in children, and shigella diarrhoea and associated stunting deaths caused an estimated 63 100 (44 000–81 900) deaths in 1 year (Table 2, Table 3). The highest burden due to ETEC and shigella infections was in the African Region (AFRO), where estimated deaths were greater than 25 000 for each pathogen (figure 1). Across WHO regions, annual ETEC mortality in children ranged from 17·8 (11·8–24·0) deaths per 100 000 children in AFRO to 1·9 (1·2–2·5) in EURO (table 2). Annual shigella mortality ranged from 34·4 (24·2–44·4) in the Eastern Mediterranean Region (EMRO) to 0·06 (0·04–0·08) deaths per 100 000 children in the European Region (EURO; table 3).

**Table 2:** Estimated ETEC burden in children younger than 5 years in low-income and lower middle-income countries

	**AFRO**	**AMRO**	**EMRO**	**EURO**	**SEARO**	**WPRO**	**Global**
**Demographics**							
Countries included in analysis	38	6	11	7	9	8	79
Population of children younger than 5 years	148 767 015	6 453 900	61 254 753	8 316 736	174 938 512	23 036 247	422 767 163
**Total deaths**							
Number of deaths (thousands)	26·5 (17·5–35·7)	0·6 (0·4–0·7)	6·6 (4·3–8·8)	0·2 (0·1–0·2)	10·0 (6·7–13·5)	0·6 (0·4–0·8)	44·4 (29·4–59·8)
Mortality rate (deaths per 100 000)	17·8 (11·8–24·0)	8·7 (5·7–11·3)	10·7 (7·1–14·4)	1·9 (1·2–2·5)	5·7 (3·8–7·7)	2·4 (1·6–3·3)	10·5 (6·9–14·1)
**Diarrhoea burden**							
Number of deaths (thousands)	21·1 (14·6–28·8)	0·5 (0·3–0·6)	5·3 (3·7–7·2)	0·1 (<0·1–0·2)	8·3 (5·8–11·4)	0·5 (0·3–0·7)	35·9 (24·7–48·9)
Mortality rate (deaths per 100 000)	14·2 (9·8–19·4)	7·5 (5·0–10·0)	8·7 (6·0–11·8)	1·6 (1·1–2·1)	4·8 (3·3–6·5)	2·1 (1·4–2·9)	8·5 (5·9–11·6)
Morbidity (millions of episodes)	36·8 (24·7–51·9)	2·3 (1·5–3·2)	13·4 (9·0–18·9)	1·5 (1·0–2·1)	27·1 (18·2–38·2)	3·3 (2·2–4·6)	84·4 (56·6–119·0)
Morbidity rate (episodes per 1000)	248 (166–349)	355 (238–501)	219 (147–309)	181 (121–255)	155 (104–218)	142 (95–200)	200 (134–281)
DALYs (thousands)	1822 (1258–2480)	44 (29–58)	464 (320–631)	13 (9–18)	735 (508–1001)	45 (31–61)	3122 (2157–4247)
DALYs (per 100 000)	1225 (846–1667)	677 (456–898)	757 (523–1029)	155 (107–211)	420 (291–572)	195 (134–264)	738 (510–1005)
**Burden from other infectious disease-related deaths due to ETEC-Induced stunting**							
Number of deaths (thousands)	5·4 (1·8–8·4)	0·1 (<0·1–0·1)	1·2 (0·4–1·9)	0·02 (0·01–0·04)	1·7 (0·6–2·6)	0·1 (<0·1–0·1)	8·5 (2·9–13·2)
Mortality rate (deaths per 100 000)	3·6 (1·2–5·6)	1·2 (0·4–1·8)	2·0 (0·7–3·1)	0·3 (0·1–0·4)	1·0 (0·3–1·5)	0·3 (0·1–0·5)	2·0 (0·7–3·1)
Increase in ETEC-related burden due to ETEC-induced stunting	26% (9–36)	16% (6–23)	23% (8–33)	18% (7–26)	20% (7–29)	17% (6–24)	24% (8–34)
Additional cases of moderate-to-severe stunting (thousands)	569 (191–894)	30 (10–47)	202 (68–317)	20 (7–30)	462 (155–724)	42 (14–66)	1325 (444–2079)
Additional cases of severe stunting (thousands)	299 (100–470)	13 (4–21)	110 (37–172)	9 (3–14)	251 (84–394)	18 (6–28)	700 (235–1098)

Data are episodes (95% UI). Episodes are the estimated number of diarrhoeal cases that children experience annually. ETEC=enterotoxigenic *Escherichia coli*. AFRO=African region. AMRO=Region of the Americas. EMRO=Eastern Mediterranean Region. EURO=European Region. SEARO=Southeast Asian Region. WPRO=Western Pacific Region.

**Table 3:** Estimated shigella burden in children younger than 5 years in low-income and lower middle-income countries

	**AFRO**	**AMRO**	**EMRO**	**EURO**	**SEARO**	**WPRO**	**Global**
**Demographics**							
Countries included in analysis	38	6	11	7	9	8	79
Population of children under 5 years of age	148 767 015	6 453 900	61 254 753	8 316 736	174 938 512	23 036 247	422 767 163
**Total deaths**							
Number of deaths (thousands)	30·1 (20·9–39·3)	0·6 (0·4–0·7)	21·1 (14·8–27·2)	<0·1 (<0·1–<0·1)	11·2 (7·9–14·6)	<0·1 (<0·1–<0·1)	63·1 (44·0–81·9)
Mortality rate (deaths per 100 000)	20·3 (14·1–26·4)	9·2 (6·3–11·6)	34·4 (24·2–44·4)	0·06 (0·04–0·08)	6·4 (4·5–8·3)	0·2 (0·1–0·2)	14·9 (10·4–19·4)
**Diarrhoea burden**							
Number of deaths (thousands)	23·1 (16·9–30·1)	0·5 (0·4–0·6)	16·8 (12·2–21·9)	<0·1 (<0·1–<0·1)	9·0 (6·6–11·8)	<0·1 (<0·1–<0·1)	49·4 (36·1–64·5)
Mortality rate (deaths per 100 000)	15·5 (11·3–20·3)	7·7 (5·5–9·8)	27·4 (20·0–35·7)	<0·1 (<0·1–0·1)	5·2 (3·8–6·7)	0·1 (<0·1–0·2)	11·7 (8·5–15·3)
Morbidity (millions of episodes)	41·3 (29·2–55·5)	2·4 (1·7–3·2)	38·1 (27·0–51·3)	<0·1 (<0·1–0·1)	29·4 (20·8–39·5)	0·2 (0·1–0·3)	111·4 (78·8–149·9)
Morbidity rate (episodes per 1000)	277 (196–373)	365 (258–491)	623 (441–838)	5·6 (4·0–7·5)	168 (119–226)	8·8 (6·2–11·8)	264 (186–355)
DALYs (thousands)	1993 (1456–2600)	45 (32–57)	1458 (1065–1901)	0·4 (0·3–0·5)	797 (584–1042)	2·8 (2·0–3·6)	4297 (3140–5604)
DALYs (per 100 000)	1340 (979–1748)	695 (496–883)	2381 (1739–3103)	4·8 (3·5–6·3)	456 (334–595)	12·1 (8·8–15·7)	1016 (743–1326)
**Burden from other infectious disease-related deaths due to shigella-Induced stunting**							
Number of deaths (thousands)	7·0 (2·4–10·7)	0·1 (0·0–0·1)	4·3 (1·5–6·4)	<0·1 (<0·1–<0·1)	2·2 (0·7–3·3)	<0·1 (<0·1–<0·1)	13·6 (4·6–20·7)
Mortality rate (deaths per 100 000)	4·7 (1·6–7·2)	1·4 (0·5–2·2)	7·0 (2·4–10·5)	<0·1 (<0·1–<0·1)	1·3 (0·4–1·9)	<0·1 (<0·1–<0·1)	3·2 (1·1–4·9)
Increase in shigella-related burden due to shigella-induced stunting	31% (11–43)	19% (7–27)	26% (9–36)	22% (8–31)	24% (9–34)	20% (7–29)	28% (10–39)
Additional cases of moderate-to-severe stunting (thousands)	748 (254–1151)	37 (12–56)	753 (256–1159)	0·7 (0·2–1·0)	603 (205–927)	3 (1–5)	2144 (728–3299)
Additional cases of severe stunting (thousands)	392 (133–603)	16 (5–25)	411 (140–632)	0·3 (0·1–0·5)	327 (111–502)	1 (<1–2)	1147 (390–1765)

Data are episodes (95% UI). Episodes are the estimated number of diarrhoeal cases that children experience annually. AFRO=African region. AMRO=Region of the Americas. EMRO=Eastern Mediterranean Region. EURO=European Region. SEARO=Southeast Asian Region. WPRO=Western Pacific Region.

**Figure 2:**
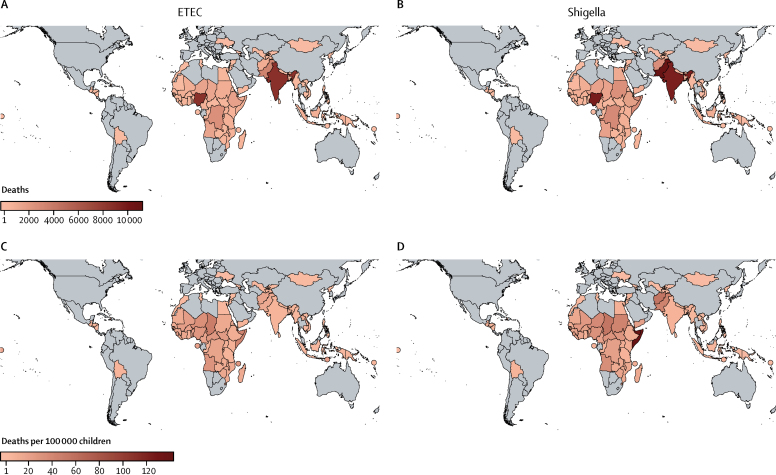
Total ETEC and shigella deaths and mortality per 100 000 children in 79 countries Estimates include deaths from direct cases of ETEC and shigella diarrhoea plus other infectious disease deaths caused by ETEC-induced and shigella-induced stunting. ETEC=enterotoxigenic *Escherichia coli*.

We estimate that more than 50% of total ETEC and shigella-related deaths were in Democratic Republic of Congo, India, Nigeria, and Pakistan (figure 2). This finding was due to high diarrhoeal mortality in these countries and large populations of children. Mortality was highest in Somalia, owing to high diarrhoeal mortality and high proportions of stunted children (figure 2). ETEC mortality was highest for countries in sub-Saharan Africa, particularly Chad, Niger, and Angola (figure 2). Shigella mortality was highest in the EMRO countries of Afghanistan and Pakistan and Chad in AFRO (figure 2).

ETEC directly caused 35 900 (95% UI 24 700–48 900) deaths per year (table 2). 21 100 (59%) deaths occurred in AFRO countries, with EURO having the fewest number of ETEC-related deaths (100 [<0·01%]). The estimated ETEC diarrhoeal mortality rate in AFRO was 1·6 times higher than in EMRO, which had the second highest rate. Direct shigella deaths were estimated at 49 400 (36 100–64 500) deaths per year (table 3). 39 900 (81%) of 49 400 shigella-related deaths were split between AFRO and EMRO countries, with the smallest fraction in EURO (four [<0·0001%] of 49 400). Shigella diarrhoeal mortality was highest in EMRO, followed by AFRO.

Together, ETEC and shigella caused 196 million (135–269) episodes of diarrhoea per year in LMICs. ETEC caused an estimated 84·4 million (56·6–119·0) diarrhoea episodes per year (table 2). AFRO (43%) and the Southeast Asian Region (SEARO; 32%) countries had the highest percentage of ETEC episodes. ETEC morbidity rates were highest in the Region of the Americas (AMRO) and AFRO countries (table 2). Shigella diarrhoea caused 111·4 million (78·8–149·9) episodes per year (table 3). Most shigella episodes occurred in AFRO (37%), EMRO (34%), and SEARO (26%) countries. Shigella diarrhoeal morbidity was greatest in EMRO, followed by AMRO (table 3).

The 79-country aggregate projections of shigella burden (4300 [3100–5600] thousand DALYs; table 3) were higher than the projected ETEC burden (3100 [2200–4200] thousand DALYs; table 2); this trend was reflected in AFRO, EMRO, and SEARO. The DALY burden of ETEC was higher than that of shigella in EURO and the Western Pacific Region (WPRO) regions, with few shigella DALYs in EURO and WPRO countries. ETEC-related DALY burden was greatest in AFRO, followed by EMRO and SEARO (table 2); shigella-related DALY burden was greatest in EMRO, followed by AFRO and AMRO (table 3). The lowest estimates of DALY burden were in EURO countries.

In aggregated estimates across 79 countries, ETEC moderate-to-severe diarrhoea episodes induced an additional 8500 [2900–13 200] child deaths in 1 year from other infectious diseases and accounted for 1·3 [0·4–2·1] million cases of moderate-to-severe stunting (table 2). About 64% of ETEC-induced stunting deaths from infectious diseases were in AFRO (5·4 [1·8–8·4] thousand deaths), with the fewest in EURO (0·2 [0·01–0·04] thousand deaths). AFRO also had the highest ETEC-induced other infectious disease mortality rate (3·6 [1·2–5·6] deaths per 100 000 children), followed by EMRO (2·0 [0·7–3·2]) and AMRO (1·2 [0·4–1·8]). ETEC-induced stunting increased the total ETEC burden estimates by 24% [8–34] for 79 countries. Other infectious disease-related deaths accounted for 17–20% of the total ETEC burden in AFRO, EMRO, and SEARO (figure 1).

Shigella-related moderate-to-severe diarrhoea episodes induced an estimated 2100 (700–3300) thousand cases of moderate-to-severe stunting and 13 600 (4600–20 700) annual child deaths from infectious diseases, with 52% (7000 of 13 600 deaths) occurring in AFRO (table 3) and low mortality in EURO (<0·01%). Shigella-induced mortality from infectious diseases were the highest in EMRO and AFRO (table 3). Shigella-induced stunting was responsible for increasing total burden by 28% (10–39) for 79 countries, accounting for 20–23% of the total shigella burden in AFRO, EMRO, and SEARO (figure 1). Diarrhoeal burden associated with shigella was much lower than ETEC in EURO and WPRO (table 3). DALY rates for ETEC were about 32 times higher than that of shigella in EURO and 16 times higher than that of shigella in WPRO.

**Figure 3:**
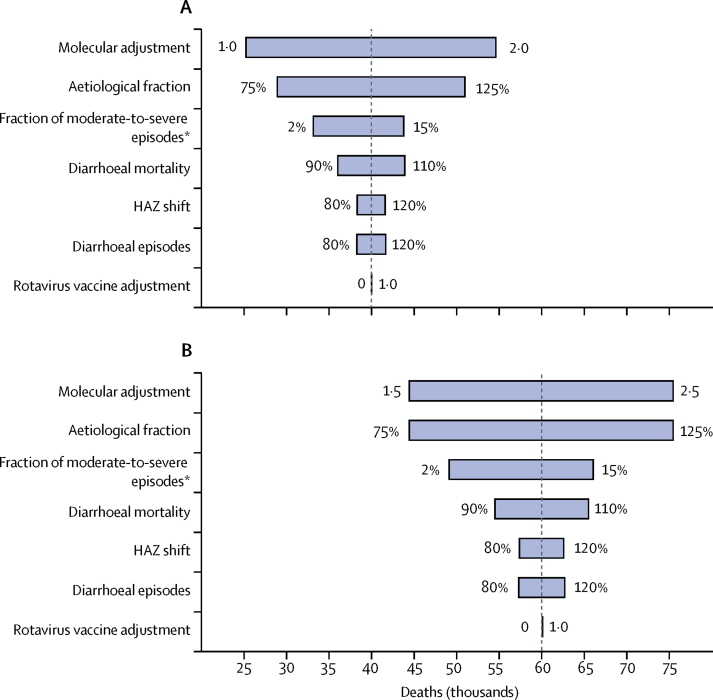
Sensitivity analyses of key input variables on the total burden of enterotoxigenic *Escherichia coli* (A) and shigella (B) disease HAZ shift is the fraction of diarrhoeal episodes attributable to enterotoxigenic *Escherichia coli* and shigella that shift HAZ scores. Rotavirus vaccination mortality adjustment accounts for the effect of the introduction of rotavirus vaccination in countries before 2014. Table 1 shows assumptions and baseline estimates. HAZ=height-for-age Z score. *Refers to the assumption that 10% of diarrhoeal episodes were considered moderate to severe and thus affected the HAZ shift.

Variables that had the largest effect on total ETEC and shigella burden in 79 countries were the molecular adjustments, aetiological fraction attributable to each pathogen, and the fraction of moderate-to-severe diarrhoea episodes causing a shift in HAZ (figure 3). Variation in burden from molecular adjustments caused swings of 56% (for ETEC mortality) and 40% (for shigella mortality) in base case estimates; these values were 31% (ETEC) and 40% (shigella) for aetiological fraction, and 7% (ETEC) and 12% (shigella) for fraction of moderate-to-severe diarrhoea episodes. Adjusting mortality estimates to account for countries that introduced rotavirus vaccination had little effect on estimates of ETEC or shigella burden.

## Discussion

The estimates from this modelling analysis show that there is a substantial unseen, and previously unquantified, morbidity and mortality resulting from non-fatal ETEC and shigella diarrhoea episodes. Among the 79 countries included in this analysis, we estimated that 200 million childhood episodes of ETEC and shigella diarrhoea occurred in 1 year. As a consequence of these infections, we further estimated that there were nearly 1·3 million cases of moderately-to-severely stunted children from ETEC episodes and 2·1 million cases from shigella episodes in 1 year. Five in ten of these malnourished children are severely stunted and at the highest risk of mortality. We estimate 8500 deaths from pneumonia, malaria, measles, and diarrhoeal disease due to ETEC and 13 600 deaths due to shigella-associated stunting. These estimates represent increases of 24% (for ETEC) and 28% (for shigella) over direct deaths from episodes, and an increase of 24% (for ETEC) and 28% (for shigella) in DALY burden. Compared with our findings, Troeger and colleagues^33^ reported increased magnitudes of effect on Global Burden of Disease estimates of diarrhoeal burden, with long-term sequalae increasing global estimates of DALY burden in children by 39·0%, with higher increases for Sub-Saharan Africa (44·1%), lower income countries in Latin America and the Caribbean (69·9%) and Southeast Asia, east Asia, and Oceana (41·0%).

There is a growing body of evidence that repeated, non-fatal infections from ETEC and shigella and other enteric pathogens contribute to a triple burden of disease from early childhood stunting.^6^ In addition to stunting-related infectious disease mortality, childhood stunting is associated with reduced cognitive development leading to poorer educational outcomes, reduced wages, and increased risk of non-communicable diseases as adults.^3^ Estimates of burden capturing these long-term consequences of ETEC and shigella infection add further value and urgency to the provision of access to safe water, sanitation, and sustained hygiene practices and future development of enteric vaccines.^12^, ^34^

ETEC-associated and shigella-associated stunting was not uniform across regions. For ETEC episodes, the highest frequencies of stunting were in AMRO and AFRO, whereas EMRO had the highest frequency of stunting due to shigella cases. The highest number of ETEC and shigella stunting-associated deaths were in AFRO. Burden due to these diseases varies across WHO regions for several reasons. First, there are regional differences in diarrhoea incidence and aetiological fractions for the two pathogens. Second, overall diarrhoeal mortality was highest in AFRO, followed by SEARO and EMRO countries. Regional differences might also be driven by nutritional deficits and country-specific risks associated with other infectious diseases. In countries with high stunting rates and a mean HAZ greater than −2, a shift in HAZ pushes more children below thresholds for moderate-to-severe stunting.

Intra-country differences in risk for disease and mortality are probably present^35^ and were not captured here. GEMS data showed variation in ETEC and shigella incidence within regions and sites based on environmental conditions and household hygiene behaviours.^10^ As one country might contain areas with no disease and very high-risk areas, quantification of intra-country heterogeneity in burden is crucial for appropriate allocation of resources. Such quantification can be done by developing risk indices that could be used to infer disease frequency. Ideally, these indices would be tested using surveillance or clinical trial data and be refined to improve predictability.

Although our analysis is based on the best available country and regional estimates, it still has several limitations. First, active surveillance providing longitudinal data at subnational scales is needed to validate current model assumptions about aetiology of diarrhoeal diseases and associated morbidity, mortality and stunting burden. Second, there is uncertainty and scarce data on the association between ETEC and shigella cases and the magnitude of the shift in HAZ. GEMS is one of the few studies quantifying an association between diarrhoea and shift in HAZ; however, it does not report the effects of duration or recurrence of diarrhoeal episodes on HAZ. Third, the incidence reported here are described as clinically-relevant diarrhoea episodes, calculated from a review of studies employing active community surveillance.^19^ Because GEMS defined the percentage of moderate-to-severe diarrhoea cases among children who presented to a clinic for diarrhoea treatment (passive surveillance), to obtain the percentage of moderate-to-severe diarrhoea in a community we adjusted the GEMS value for the proportion of children who are likely to be brought for diarrhoea treatment. We calculated that 10% of episodes were moderate-to-severe cases^10^ and, as the percentage of children with moderate-to-severe diarrhoea and the percentage of children with diarrhoea who could seek care might vary by place and time, used a sensitivity analysis to assess variation of 2–15% moderate-to-severe diarrhoea on disease burden. As expected, the percentage of moderate-to-severe diarrhoea cases had a substantial effect on ETEC and shigella burden. Fourth, our study was affected by the paucity of knowledge of the relative risk of dying from other infectious diseases when stunted. The relative risk estimates we used in our model were calculated before widespread use of pneumococcal, rotavirus, and measles vaccines, which affects the relative risk of mortality. Fifth, we applied molecular adjustments to culture-based estimates of aetiological fractions of ETEC and shigella mortality and morbidity. Molecular studies report increased detection of ETEC, shigella, and similar pathogens in gastrointestinal infections,^23^, ^24^, ^25^ but few studies quantify correction values (as was done in the GEMS reanalysis study). Our sensitivity analysis showed that variation in this assumption affected shigella estimates more than ETEC, but was the most influential assumption on total burden estimates for both pathogens. Finally, our analysis focused on ETEC and shigella disease in children younger than 5 years. However, evidence from studies of children attending primary school supports the hypothesis that older children are also at high risk for diarrhoeal diseases.^36^, ^37^ Future studies should quantify the burden of diarrhoeal diseases in this age group.

In summary, our findings show that mortality estimates from ETEC and shigella together with deaths from infection-associated stunting results in a substantially increased mortality burden than previously considered. Our results show the importance of quantifying short-term and long-term effects of infections to understand the true disease burden. Here, we have captured a subset of the potential effects of non-fatal episodes; there is also evidence linking childhood stunting to a series of other important health and economic outcomes throughout life, including reduced cognitive function, educational attainment, earnings, and chronic diseases.^3^, ^6^ Improved quantification of all of the effects of infection and treatment costs, including the potential for treatment regimens to become less effective as a result of anti-microbial resistance, is necessary to better describe the full effect of potential ETEC and shigella interventions. This includes the potential for vaccines to show their overall cost-effectiveness by preventing infection, infection associated life-threatening illness, and socioeconomic consequences of morbidity.
