# Supplementary material for: Burden of enterotoxigenic Escherichia coli and shigella non-fatal diarrhoeal infections in 79 low-income and lower middle-income countries: a modelling analysis
Source: Lancet Glob Health. 2019 Feb 14;7(3):e321–30. doi: 10.1016/S2214-109X(18)30483-2 (PMC6379821; doi:10.1016/S2214-109X(18)30483-2)
Supplement: Supplementary appendix [file mmc1.pdf]

THE LANCET
Global Health

Supplementary appendix

This appendix formed part of the original submission and has been peer reviewed. We post it as supplied by the authors.

Supplement to: Anderson IV JD, Bagamian KH, Muhib F, et al. Burden of enterotoxigenic *Escherichia coli* and shigella non-fatal diarrhoeal infections in 79 low-income and lower middle-income countries: a modelling analysis. *Lancet Glob Health* 2019; **7:** e321–30.

Contents

Page 1: Supplemental figure 1 – Conceptual and analytical framework

Page 2: Supplemental table 1 – The 79 countries included in the study, by WHO region

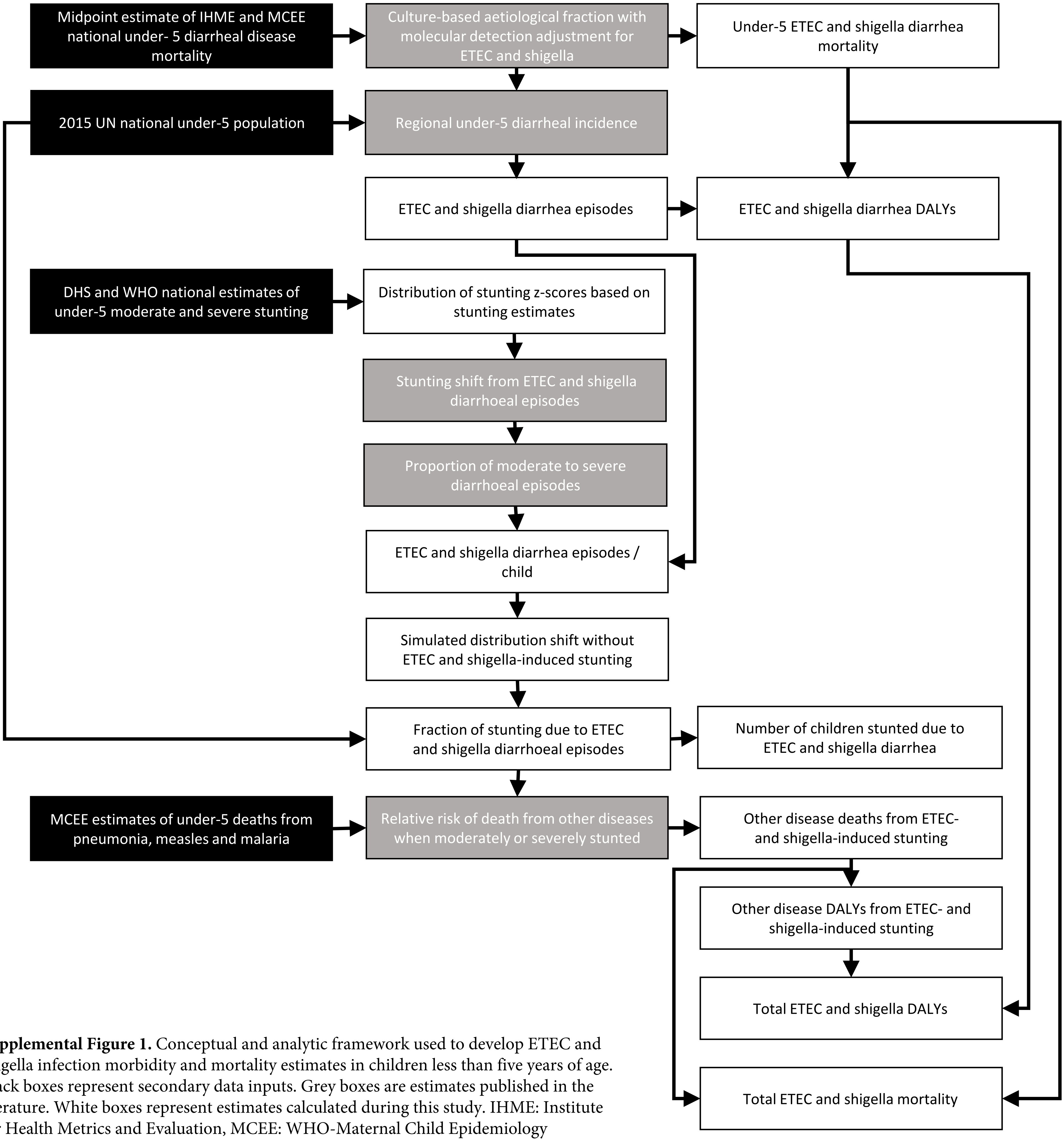

Supplemental Figure 1. Conceptual and analytic framework used to develop ETEC and shigella infection morbidity and mortality estimates in children less than five years of age. Black boxes represent secondary data inputs. Grey boxes are estimates published in the literature. White boxes represent estimates calculated during this study. IHME: Institute for Health Metrics and Evaluation, MCEE: WHO-Maternal Child Epidemiology Estimation, DHS: Demographic and Health Surveys.

Supplemental Table 1. The 79 countries included in the study by WHO region. AFRO: African region, AMRO: Region of the Americas, EMRO: Eastern Mediterranean Region, SEARO: Southeast Asian Region, WPRO: Western Pacific Region. *Denotes low-income countries, all others are lower middle-income countries

AFRO		AMRO	EMRO	EURO	SEARO	WPRO	Excluded
Angola	Liberia*	Bolivia	Afghanistan*	Armenia	Bangladesh	Cambodia	Cabo Verde
Benin*	Madagascar*	El Salvador	Djibouti	Georgia	Bhutan	Kiribati	Kosovo
Burkina Faso*	Malawi*	Guatemala	Egypt	Kyrgyz Republic	DPR Korea*	Lao PDR	Micronesia
Burundi*	Mali*	Haiti*	Jordan	Moldova	India	Mongolia	Moldova
Cameroon	Mauritania	Honduras	Morocco	Tajikistan	Indonesia	Papua New Guinea	Vanuatu
Central African Republic*	Mozambique*	Nicaragua	Pakistan	Ukraine	Myanmar	Philippines	
Chad*	Niger*		Somalia*	Uzbekistan	Nepal	Solomon Islands	
Comoros*	Nigeria		Sudan		Sri Lanka	Viet Nam	
Congo	Rwanda*		Syria		Timor-Leste		
Congo DR	São Tomé & Principe		Tunisia				
Côte d'Ivoire	Senegal*		Yemen				
Eritrea*	Sierra Leone*						
Ethiopia*	South Sudan*						
The Gambia*	Swaziland						
Ghana	Tanzania*						
Guinea*	Togo*						
Guinea-Bissau*	Uganda*						
Kenya	Zambia						
Lesotho	Zimbabwe*						
